# Metabolic engineering of the L-phenylalanine pathway in *Escherichia coli *for the production of S- or R-mandelic acid

**DOI:** 10.1186/1475-2859-10-71

**Published:** 2011-09-13

**Authors:** Zhoutong Sun, Yuanyuan Ning, Lixia Liu, Yingmiao Liu, Bingbing Sun, Weihong Jiang, Chen Yang, Sheng Yang

**Affiliations:** 1 Key Laboratory of Synthetic Biology, Institute of Plant Physiology and Ecology, Shanghai Institutes for Biological Sciences, Chinese Academy of Sciences, Shanghai, China; 2 Graduate University of Chinese Academy of Sciences, Beijing, China; 3 Shanghai Research and Development Center of Industrial Biotechnology, Shanghai, China; 4 Huzhou Center of Industrial Biotechnology, Shanghai Institutes for Biological Sciences, Chinese Academy of Sciences, Huzhou, China

**Keywords:** *Escherichia coli*, fermentation, fine chemicals, metabolic engineering, renewable resources, R-mandelic acid, S-mandelic acid

## Abstract

**Background:**

Mandelic acid (MA), an important component in pharmaceutical syntheses, is currently produced exclusively via petrochemical processes. Growing concerns over the environment and fossil energy costs have inspired a quest to develop alternative routes to MA using renewable resources. Herein we report the first direct route to optically pure MA from glucose via genetic modification of the L-phenylalanine pathway in *E. coli*.

**Results:**

The introduction of hydroxymandelate synthase (HmaS) from *Amycolatopsis orientalis *into *E. coli *led to a yield of 0.092 g/L S-MA. By combined deletion of competing pathways, further optimization of S-MA production was achieved, and the yield reached 0.74 g/L within 24 h. To produce R-MA, hydroxymandelate oxidase (Hmo) from *Streptomyces coelicolor *and D-mandelate dehydrogenase (DMD) from *Rhodotorula graminis *were co-expressed in an S-MA-producing strain, and the resulting strain was capable of producing 0.68 g/L R-MA. Finally, phenylpyruvate feeding experiments suggest that HmaS is a potential bottleneck to further improvement in yields.

**Conclusions:**

We have constructed *E. coli *strains that successfully accomplished the production of S- and R-MA directly from glucose. Our work provides the first example of the completely fermentative production of S- and R-MA from renewable feedstock.

## Background

Mandelic acid (MA), an important fine chemical, has been widely used in the synthesis of cephalosporin antibiotics [1], the preparation of various other pharmaceuticals [2-4], as well as the resolution of racemic alcohols and amines [5,6]. Its stereoisomers generally are prepared using a chemical approach [7-9]. However, it has also been reported that its stereoisomers can be prepared via enzymatic routes; for example, stereospecific nitrilases are used to produce S- or R-MA [10-13] and enantioselective microbes are employed to degrade S-MA or R-MA to yield R- or S-enantiomers [14-16], respectively. In addition, a system combining the two microbes *Pseudomonas polycolor *and *Micrococcus freudenreichii *has also been explored for the production of R-MA from the racemate [17]. However, these commodity chemicals are manufactured entirely from petroleum-based feedstock such as benzaldehyde and mandelonitrile. Growing concerns over the environment and fossil resources costs have inspired a quest to develop more sustainable processes that afford these products from renewable feedstock at lower cost. Thus, biological processes based on renewable resources such as glucose that would provide direct one-step production of chiral MA in a microbial fermentation process would be of commercial interest.

Thus far, no direct biosynthetic pathway to free chiral MA has been identified in nature, although some organisms possess an MA catabolic pathway [18]. Engineering a microbe for the production of a heterologous compound requires the establishment of a new biochemical pathway. To construct synthetic pathways for producing S-MA and R-MA in *E. coli*, two problems need to be resolved. Firstly, suitable starting intermediates must be available. Secondly, the introduction of enzymes catalyzing reactions to produce the desired products must be feasible.

**Figure 1:**
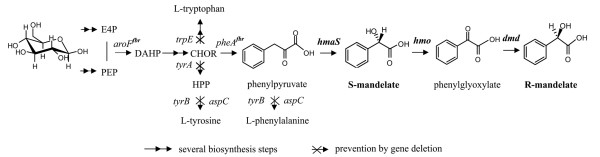
**Schematic representation of S-MA or R-MA biosynthesized from glucose in engineered *E. coli***. ***hmaS*: **Hydroxymandelate synthase gene from *A. orientalis*; ***hmo*: **Hydroxymandelate oxydase gene from *S. coelicolor*; ***dmd*: **D-mandelate dehydrogenase gene from *R. graminis*; **E4P: **erythrose-4-phosphate; **PEP: **phosphoenolpyruvate; **CHOR: **chorismate; **HPP: **hydroxyphenylpyruvate; **DAHP: **3-deoxy-D-arabino-heptulosonate-7-phosphate.

Here we describe the construction of synthetic pathways for the production of S-MA and R-MA in *E. coli *by combining biological entities from various organisms. For the production of S-MA, 4-hydroxymandelate synthase (HmaS) from *Amycolatopsis orientalis *was used to convert phenylpyruvate to S-MA [19]. To expand the S-MA synthesis to produce R-MA, two other enzyme reactions were explored: 4-hydroxymandelate oxidase (Hmo) from *Streptomyces coelicolor *was used to convert S-MA to phenylglyoxylate [19] and a third enzyme, D-mandelate dehydrogenase (DMD) derived from *R. graminis*, was used for the stereo-reduction of phenylglyoxylate to R-MA [20]. As phenylpyruvate is the direct precursor of L-phenylalanine, starting from phenylpyruvate it should be possible to construct an artificial pathway using HmaS, Hmo, and DMD to biosynthesize chiral MA directly from glucose in *E. coli *(Figure 1).

In this study, we have modified *E. coli *strains that successfully achieved the production of S- and R-MA directly from glucose. To our knowledge, this is the first report describing the engineering of the L-phenylalanine pathway in *E. coli *to produce enantiomerically pure S-MA and R-MA directly from a renewable resource such as glucose.

## Results

### Fermentation of glucose via expression of HmaS in *E. coli *to produce S-MA directly

In order to construct a pathway to produce S-MA via a modified L-phenylalanine pathway in *E. coli*, an enzyme with S-mandelate synthesis activity is required to convert phenylpyruvate to S-MA. Thus, HmaS from *A. orientalis *was cloned and over-expressed in *E. coli*; the specific activity of HmaS was determined to be 10.18 mU/mg total protein. Production of MA in the reaction broth was identified by HPLC equipped with a chiral column, and the results illustrated that HmaS was able to convert phenylpyruvate to S-MA (Additional file 1), but not for the control (Additional file 1). Surprisingly, trace amounts of R-MA were detected as well, the results revealing that HmaS is not a rigorous stereo-inverting enzyme for catalyzing phenylpyruvate to S-MA. The enantiomeric excess (ee), > 98%, was calculated according to a previous report [15]. Therefore, this enzyme could be used further for the preparation of S-MA in *E. coli*.

**Figure 2:**
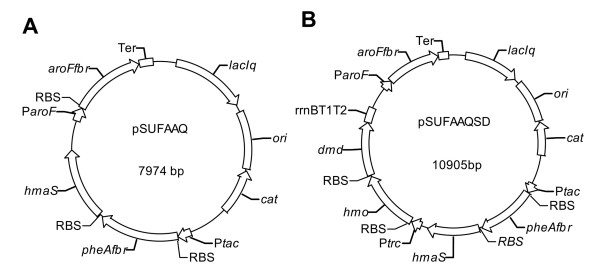
**Recombinant plasmids constructed in this study**. **(A) **The recombinant plasmid pSUFAAQ used for S-MA synthesis. **(B) **The recombinant plasmid pSUFAAQSD used for R-MA synthesis.

Over-expressions of deregulated 3-deoxy-D-arabino-heptulosonate-7-phosphate (DAHP) synthase and chorismate mutase/prephenate dehydratase are well known strategies for increasing L-phenylalanine production in *E. coli *[21]. In this study, both the feedback deregulated DAHP synthase encoded by *aroF^fbr ^*[22] and chorismate mutase/prephenate dehydratase encoded by *pheA^fbr ^*[23] were constructed and over-expressed to increase the carbon flow into the shikimate pathway and ensure sufficient flow from chorismate to phenylpyruvate. To allow the fermentation of glucose to produce S-MA, an artificial operon with *aroF^fbr^, pheA^fbr ^*and *hmaS *over-expression was created, which resulted in the recombinant plasmid pSUFAAQ (Figure 2A).

To produce S-MA in *E. coli*, wild-type strains of *E. coli *W3110 harboring pSUFAAQ were cultured in fermentation medium in shake flasks. This resulted in the production of 0.092 g/L S-MA within 24 h, and a large amount of L-phenylalanine was detected as well. This proved that the artificial operon was functionally expressed in *E. coli*. Therefore, this strain could be used as a basis strain for further improvement to the production of S-MA directly from glucose. The biosynthetic pathway for S-MA is shown schematically in Figure 1.

### Combined deletion of competing pathways to optimize the production of S-MA

**Figure 3:**
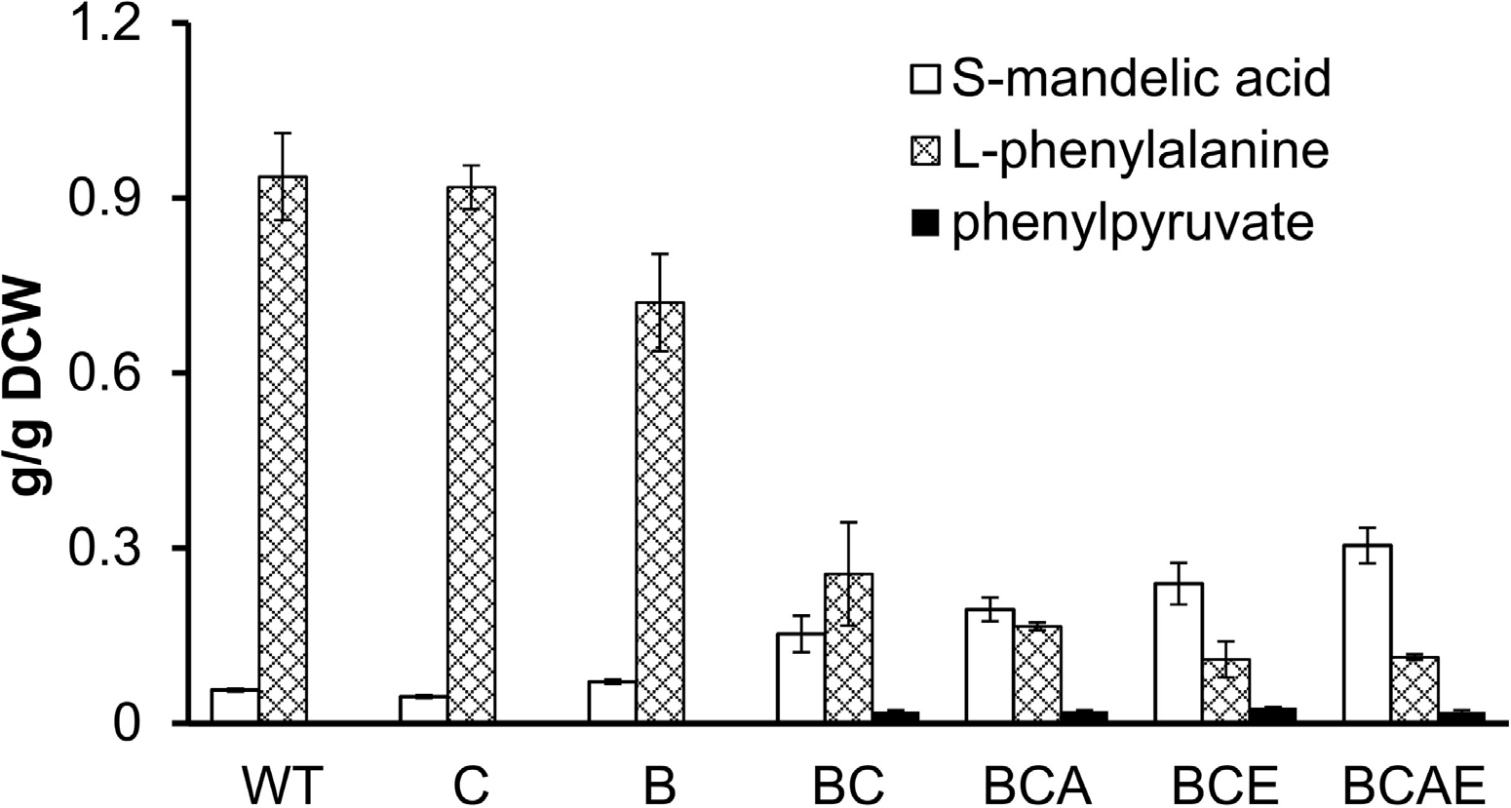
**Biosynthesis of S-MA in different mutants with pSUFAAQ**. Different deletion mutants containing recombinant pSUFAAQ were cultivated in shake flasks for 24 h. The product S-mandelic acid, the intermediate phenylpyruvate, and the byproduct L-phenylalanine were analyzed. Data shown are means ± standard deviations, calculated from triplicate individual experiments.

To further to optimize S-MA production, one logical possibility is the reduction of the byproducts formed. Thus, genes involved in competing side reactions would need to be deleted to avoid the consumption or degradation of the desired intermediates. Since the concentration of phenylpyruvate was the major limitation in the synthesis of S-MA, the aromatic amino acid aminotransferase encoded by *tyrB *and aspartate aminotransferase encoded by *aspC*, which are involved in the conversion of phenylpyruvate to L-phenylalanine, were deleted to minimize the loss of phenylpyruvate. The results showed that the deletion of *tyrB *increased S-MA production (0.07 g/gDCW) in comparison to the wild-type (0.06 g/gDCW), whereas the deletion of *aspC *had no positive effect on S-MA production (0.05 g/gDCW) and produced 0.92 g/gDCW L-phenylalanine. Furthermore, the production of S-MA significantly increased up to 0.15 g/gDCW (a 2.5-fold increase compared to wild-type), whereas the byproduct of L-phenylalanine (0.26 g/gDCW) was dramatically reduced in double mutants (Figure 3).

To further increase the carbon flux to phenylpyruvate, the branch pathways of L-tyrosine and L-tryptophan were disrupted based on *tyrB *and *aspC *double mutants (BC) to reduce divergence from chorismate. Then we deleted the chorismate mutase/prephenate dehydrogenase encoded by *tyrA *and the anthranilate synthase encoded by *trpE*. As expected, for *tyrA *or/and *trpE *deletion based on double mutants, the production of S-MA increased to 0.30 g/gDCW (BCAE) with a 2-fold increase compared to BC and a 5-fold increase compared to wild-type.

We still detected amounts of phenylpyruvate (approximately 0.02 g/gDCW) that accumulated from BC to BCAE (Figure 3), which implied that the activity of HmaS was insufficient to convert all precursors of phenylpyruvate to S-MA. We postulate that combined deletion of the competing pathways led to increased phenylpyruvate accumulation and resulted in driving HmaS to synthesize more S-MA. Therefore, HmaS might be a potential bottleneck for further optimization of S-MA production and its derivatives.

### Characterization of the fermentation of recombinant strain BCAE/pSUFAAQ

**Figure 4:**
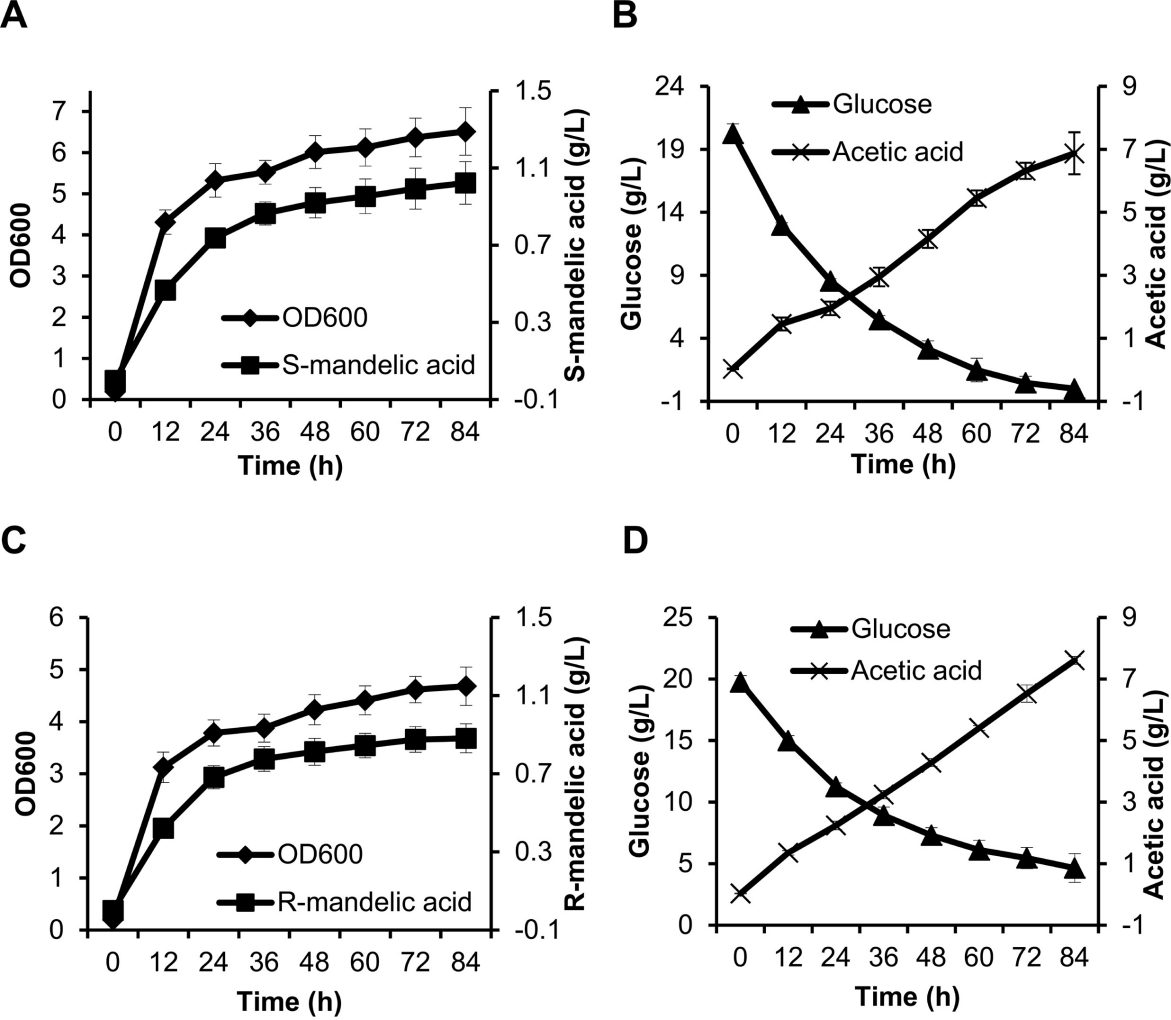
**Time profiles of cell growth, S- or R- mandelic acid production, and residual glucose and acetic acid accumulation during the culture of the engineered *E. coli***. **(A) **OD_600_, S-mandelic acid produced, and **(B) **glucose consumed, byproduct acetic acid accumulated were monitored for BCAE with pSUFAAQ. **(C) **OD_600_, R-mandelic acid produced, and **(D) **glucose consumed, byproduct acetic acid accumulated were monitored for BCAE with pSUFAAQSD. Data shown are means ± standard deviations calculated from triplicate individual experiments.

To further characterize the potential capacity of the best strain of BCAE harboring pSUFAAQ for S-MA production, shake flask fermentation was performed. During the process of fermentation, the cell density, S-MA production, glucose assimilation, and byproduct acetic acid accumulation were monitored, as shown in Figure 4A and 4B. As a result, 0.74 g/L S-MA was observed within 24 h, with a yield of 6.5% (*g/g*) from glucose, which reached 31% of the theoretical maximum (theoretical yield is 21% from glucose). The maximum production of S-MA reached 1.02 g/L within 84 h (Figure 4A) and, at the same time, glucose was completely consumed (Figure 4B). The optical isomer of MA in the broth was identified by HPLC using a chiral column (Additional file 2), confirming the formation of the S-isomer. Then the products in the broth were further analyzed by GC-MS (Additional file 3), confirming the product produced by recombinant strain BCAE/pSUFAAQ is S-MA. Therefore, the strain BCAE/pSUFAAQ could be used as the potential production strain in further studies.

In addition, 1.95 g/L of the byproduct acetic acid was detected in 24 h, and the titer significantly increased to 6.87 g/L within 84 h (Figure 4B). Acetic acid accumulation could cause many obstacles to the industrial production of interesting products. To minimize acetate formation, many strategies have been developed for this purpose during *E. coli *aerobic fermentations [24].

### Use of Hmo together with DMD to convert S-MA via phenylglyoxylate to R-MA *in vitro*

Thus far, no enzyme with R-mandelate synthase activity has been identified that could catalyze the precursor of the amino acid to R-MA. Therefore, to promote fermentation production of R-MA *in vivo*, two other enzymes are required to convert S-MA via phenylglyoxylate to R-MA. In this study, Hmo from *S. coelicolor *and DMD from *R. graminis *were cloned and expressed in *E. coli*. Then, the specific activities of Hmo and DMD were determined to be 0.257 mU/mg and 87.44 mU/mg, respectively. These enzymes were used for further studies.

To test whether Hmo together with DMD could transform S-MA to R-MA *in vitro*, these two genes were cloned into a vector of pTrc99a under IPTG inducible promoter *P*_trc _resulting in pTrc99aSD. Then an assay for the simultaneous catalysis of S-MA to R-MA via the enzymes Hmo and DMD was performed *in vitro*. As shown in Additional file 1, R-MA was detected, but not for the control (Additional file 1). Thus, the conversion of S-MA via phenylglyoxylate to R-MA proved the functional expression of these two enzymes in *E. coli*, and paved the way for further synthesis of R-MA using their activities *in vivo*.

### Co-expression of *hmaS, hmo*, and *dmd *in engineered *E. coli *to produce R-MA directly from glucose fermentation

We have demonstrated that expression of HmaS in *E. coli *can ferment glucose to produce S-MA and proved that the simultaneous expression of Hmo and DMD can convert S-MA via phenylglyoxylate to R-MA *in vitro*. Therefore, we presumed that co-expression of HmaS, Hmo, and DMD in *E. coli *could produce R-MA from glucose directly.

To produce R-MA ultimately from glucose, a new artificial biosynthetic pathway was assembled in *E. coli*. The scheme of this new pathway is shown in Figure 1. Based on the findings described above, we constructed an artificial biosynthetic pathway containing HmaS, Hmo, and DMD, with the corresponding genes obtained from *A. orientalis, S. coelicolor*, and *R. graminis*, respectively. Then, an engineered *E. coli *strain BCAE with the recombinant plasmid pSUFAAQSD (Figure 2B) was created.

Batch cultivations of BCAE/pSUFAAQSD were performed in shake flasks. During the fermentation process, cell density, R-MA production, glucose assimilation, and accumulation of the byproduct acetic acid were monitored (Figure 4C and 4D). The results illustrated that 0.68 g/L of R-MA with a yield of 7.8% (*g/g*) from glucose was achieved within 24 h and reached 37% of theoretical maximum, and the titer of R-MA reached 0.88 g/L in 84 h. The fermentation broth was analyzed by HPLC equipped with a chiral column (Additional file 2), and the products were further identified by GC-MS (Additional file 4). Combining these data showed that the compound in the broth was confirmed as R-MA.

Therefore, in this study we demonstrated that combined expression of the three enzymes in engineered *E. coli *strains resulted in the first completely fermentative synthesis of R-MA directly from glucose.

### HmaS might be a potential bottleneck for further improvement of chiral MA production

**Figure 5:**
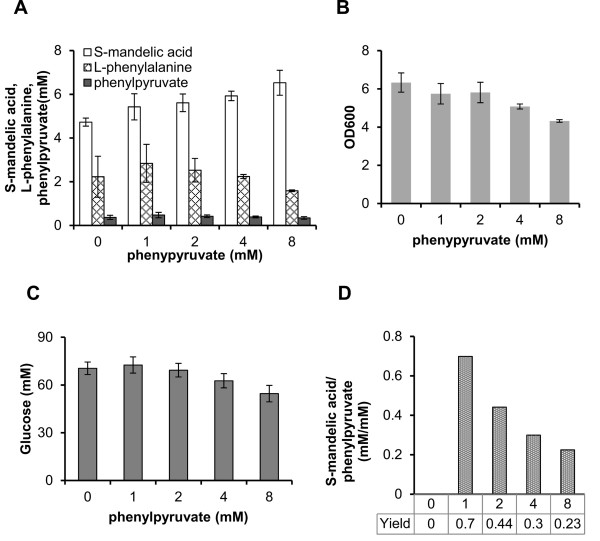
**Biosynthesis of S-mandelic acid with phenylpyruvate supplied**. Products of engineered strains BCAE with pSUFAAQ fed with various concentrations of phenylpyruvate were analyzed in shake flasks during 24 h. S-mandelic acid, L-phenylalanine, and phenylpyruvate **(A)**, cell growth **(B)**, glucose consumption **(C)**, and yields of S-mandelic acid from supplied phenylpyruvate **(D) **are listed. Data shown are means ± standard deviations calculated from triplicate individual experiments.

To further investigate HmaS as a potential rate-limiting enzyme preventing further improvement in the yields of S-MA and R-MA, we cultured BCAE with pSUFAAQ with various quantities of phenylpyruvate supplied. We found that the titers of S-MA gradually increased as the concentration of phenylpyruvate increased (Figure 5A). However, the increase in rate was not remarkable and a maximum of up to 38% was achieved with 8 mM phenylpyruvate supplied, whereas the cell density declined from OD_600 _of 6.3 to 4.3, suggesting that the higher concentrations of phenylpyruvate might be toxic to the host. It is also clear that the yield of S-MA from phenylpyruvate was obviously reduced from 0.70 (mM/mM) to 0.23 (mM/mM) with a feeding gradient of 1 mM to 8 mM (shown in Figure 5D). These results implied that the activity of HmaS was insufficient to catalyze all supplied phenylpyruvate to S-MA.

Furthermore, the kinetics parameters of HmaS were measured: the K_m _of HmaS was 0.45 ± 0.04 mM with phenylpyruvate as substrate, which was approximately 70-fold greater than the K_m _with its native substrate 4-hydroxyphenylpyruvate (6.5 ± 0.8 μM) [25]. This indicates that more phenylpyruvate is needed, compared to its native substrate, to achieve the same effect for HmaS synthesis of the corresponding products. Therefore, the K_m _of HmaS for phenylpyruvate needs to be modified to improve the catalytic efficiency at lower phenylpyruvate concentrations. This would be useful not only in terms of HmaS competing with other enzymes for catalyzing phenylpyruvate to S-MA, but would also be beneficial in alleviating phenylpyruvate toxicity towards the host at lower concentrations.

Therefore, in combining these data we infer that HmaS will be an important target for further strain improvement to enhance S-MA and R-MA production.

## Discussion

Chiral mandelic acid, an important medical intermediate, is mainly prepared by chemical or enzymatic approaches. In this study, a new fermentative route was created using an engineered *E. coli *to produce optical isomers based on renewable resources. The L-phenylalanine pathway was modified and an artificial pathway using the enzymes HmaS, Hmo, and DMD from *A. orientalis, S. coelicolor*, and *R. graminis*, respectively, was constructed.

Many enzymatic methods have been reported for the preparation of stereoisomers [10,13]. However, none of them were able previously to produce mandelate enantiomers from glucose directly in a single microbial fermentation step. Thus far, substrates of enzymatic routes such as benzaldehyde and mandelonitrile have not been identified among the microbial primary metabolic pathways. If in the future some enzymes are discovered for catalyzing the primary metabolites to benzaldehyde or mandelonitrile, such multistep enzyme-catalyzed processes might be developed in the microbe for the production of mandelate isomers from glucose. However, the conversion rate would not be superior to that in this report due to the requirement for more heterogeneous enzymes and the extra reaction steps required.

Due to a lack of R-mandelate synthase, which could catalyze phenylpyruvate to R-MA directly, a relatively complex artificial pathway was constructed in this work for the production of R-MA. If R-mandelate synthase were available, the method for preparation of R-MA described here would be optimized using R-mandelate synthase only. We tried to find this kind of enzyme in some microorganisms using bioinformatics means. Unfortunately, thus far we have not been able to find it due to limited resources; however, it should be possible to construct enzymes with R-mandelate synthase activity through directed evolution.

Although we have successfully achieved the preparation of S-MA and R-MA in engineered *E. coli*, the byproduct of L-phenylalanine was still formed by the double mutant. It could be that the branched-chain amino acid aminotransferase encoded by *ilvE *is able to catalyze phenylpyruvate to L-phenylalanine [26], so the deletion of *ilvE *should eliminate L-phenylalanine formation to optimize the production of S-MA and R-MA in the future.

We observed the accumulation of large quantities of acetic acid during the fermentation process (Figure 4B and 4D). Acetic acid excretion by *E. coli *during aerobic growth on glucose is a major obstacle to recombinant protein production and chemical products, and gene disruptions have been successfully employed to minimize acetic acid formation and increase the production level of desired chemicals [27-30]. In this work, to improve MA production, we have deleted acetate-forming genes including *poxB, pta, ackA*, and *acs *to reduce acetic acid formation. Results showed that single gene deletions did not reduce acetate accumulation, suggesting that alternative pathways were also involved in acetate production (Additional file 5). As expected, simultaneous deletion of *poxB, ackA *and *acs *greatly minimized acetate accumulation (Additional file 6). However, the acetate reduction led to no increase in the carbon flux to MA production, which might be caused by significantly reduced cell growth rates.

In order to improve the production of S-MA, two genes (*ppsA *and *tktA*) [31] were co-expressed in the engineered S-MA-producing strains to enhance the supply of precursors of PEP and E4P. In the resulting strains the productivity of S-MA improved whereas the titer did not improve (data not shown). This result indicated that some other bottleneck exists downstream of the S-MA synthesis pathway.

HmaS is not only a key enzyme for S-MA synthesis but is also the first step in R-MA synthesis. We have previously investigated the effects of different mutants on S-MA production. The results implied that the activity of HmaS was insufficient to convert all phenylpyruvate to S-MA, leading to the accumulation of phenylpyruvate in the culture. To further explore the activity of HmaS, we also conducted phenylpyruvate feeding experiments. Although higher concentrations of phenylpyruvate slightly enhanced the yield of S-MA, the conversion rate of HmaS was significantly reduced with increasing phenylpyruvate. Furthermore, we measured the kinetic parameters of HmaS and found that its affinity towards the non-natural substrate phenylpyruvate was much lower than that towards its natural substrate [25]. This suggests that, in order to synthetize S-MA, HmaS needs a higher concentration of the substrate phenylpyruvate as the driving force. Therefore, combining these data we infer that HmaS will potentially be the bottleneck for further improvement of S-MA and R-MA synthesis. Thus, the catalytic activity of HmaS for converting phenylpyruvate to S-MA should be improved first through protein engineering to further improve the titers and yields of S-MA and R-MA production.

## Conclusions

We have established a functional artificial pathway in *E. coli *and developed the first completely fermentative route for producing optically pure S-MA and R-MA from glucose, though further strain improvement and fermentative process optimization, including metabolic flux analysis, will be needed to improve the production of S-MA and R-MA. The successful construction of S-MA- and R-MA-producing *E. coli *in this study provides a solid basis for the development of other engineered bacteria, which could yield other commercially attractive bio-products or their derivatives from renewable resources.

## Methods

### Bacterial strains and culture media

Strains and plasmids used in this study are listed in Table 1. Antibiotics were used when needed at the following concentrations: Ampicillin (100 mg/L), Chloramphenicol (25 mg/L), Kanamycin (50 mg/L), Tetracycline (15 mg/L), Spectinomycin (50 mg/L), Apramycin (50 mg/L).

**Table 1:** Strains and plasmids used in this study


**Strains**	**Genotype**	**Reference**

*A. orientalis* *HCCB10007*	Vancomycin-producing bacteria	[37]
*S. coelicolor* A3(2) M145	Wild type SCP1^- ^SCP2^-^	[38]
*R. graminis* ATCC20804	*Rhodotorula graminis *di Menna, anamorph, mutant derived from KGX39	ATCC
DH5α	*lacZ*ΔM15 *endA1 recA1 relA1 gyrA96 deoR nupG *λ^-^	TaKaRa
JM105	*endA1 glnV44 sbcB15 rpsL thi-1 *Δ(lac-proAB) [F' *traD36 proAB^+ ^lacI^q ^lacZ*ΔM15] *hsdR*4 (r_K_^-^m_K_^+^)	Pharmacia
BL21(DE3)	F^- ^*omp gal dcm lon hsdS_B _*(r_B_^-^m_B_^-^) λ(DE3)	Novagen
JW1256-1	F^- ^λ^- ^Δ*lacZ4787 *(::rrnB-3), Δ*trpE::*FRT*-kan-*FRT, *rph-1 hsdR514*	CGSC
N3087	*tyrA16*::Tn10 *rpsL31*(str^R^)	CGSC
WT	Wild type *E. coli *W3110 F^- ^λ^- ^*rph-1 INV *(rrnD rrnE)	CGSC
B	W3110 Δ*tyrB*::FRT	This study
C	W3110 Δ*aspC*::FRT	This study
BC	W3110 Δ*tyrB*::FRT, Δ*aspC*::FRT	This study
BCA	W3110 Δ*tyrB*::FRT, Δ*aspC*::FRT, *tyrA16*::Tn10	This study
BCE	W3110 Δ*tyrB*::FRT, Δ*aspC*::FRT, Δ*trpE*::FRT	This study
BCAE	W3110 Δ*tyrB*::FRT, Δ*aspC*::FRT, *tyrA16*::Tn10, Δ*trpE*::FRT	This study
		
Plasmids	Genotype	Reference

pET24a	*kan P*_T7 _expression vector	Novagen
pEThmaS_Ao_	pET24a derivative, *hmaS_Ao _*from *A. orientalis *	This study
pET24admd	pET24a derivative, *dmd *from *R. graminis *	This study
pTrc99a	*bla P*trc *lacI^q ^*pBR322 ori expression vector	Pharmacia
pTrc99ahmo	pTrc99a derivative, *hmo *	This study
pTrc99aSD	pTrc99a derivative, *hmo, dmd *	This study
pKK223-3	*bla P*tac pBR322 ori expression vector	Pharmacia
pKKpheA^fbr^	pKK223-3 derivative, *pheA^fbr ^*from *E. coli *	This study
pSU2718	*Hae *II fragment of pUC18 containing *lacZα *and MCS, p15A replicon with chloramphenicol acetyl transferase gene (*cat*)	[39]
pSUaroF^fbr^	pSU2718 derivative, *aroF^fbr ^*from *E. coli*	This study
pSUFA	pSU2718 derivative, *aroF^fbr^, pheA^fbr ^*	This study
pSUFAQ	pSU2718 derivative, *aroF^fbr^, pheA^fbr^, lacI^q^*	This study
pSUFAAQ	pSU2718 derivative, *aroF^fbr^, pheA^fbr^, lacI^q^, hmaS_Ao_*	This study
pSUFAAQSD	pSU2718 derivative, *aroF^fbr^, pheA^fbr^, lacI^q^, hmaS_Ao_, hmo, dmd*	This study
pIJ790	*araC P*_araBAD _*gam bet exo repA*101^ts ^*oriR*101 *cat*	[33]
pIJ773	*bla *FRT-*aac(3)IV-oriT*-FRT	[33]
pIJ778	*bla *FRT-*aadA-oriT*-FRT	[33]
BT340	*t^s ^bla cat*	[33]

LB medium was used for culturing the general strains for cloning. The engineered *E. coli *strains were cultivated in shake flasks in fermentation medium to test S- or R-MA production *in vivo*.

The fermentation medium contained the following components: D-glucose (20 g/L), KH_2_PO_4 _(1.0 g/L), (NH_4_)_2_SO_4 _(16 g/L), MgSO_4 _7H_2_O (1.0 g/L), L-tyrosine (0.2 g/L), L-tryptophan (0.1 g/L), L-aspartic acid (3 g/L), FeSO_4 _7H_2_O (0.01 g/L), MnSO_4 _H_2_O (0.008 g/L), CaCO_3 _(20 g/L). The stock solution of D-glucose, CaCO_3_, and MgSO_4 _was autoclaved separately whereas L-tryptophan, FeSO_4_, and MnSO_4 _were sterilized using a 0.22-μm filter.

### Plasmid construction

The gene encoding feedback resistant mutant *aroF^fbr ^*P148L [22] including the native promoter was obtained by overlap PCR, and the genomic DNA of *E. coli *W3110 was used as the PCR template. Two overlapping DNA fragments were amplified using primer pairs P148L-F/aroFSacI-R and P148L-R/aroFSacI-F, respectively. Then, using the two overlapping DNA fragments as the templates, another PCR reaction was performed with primer pair aroFSacI-F/aroFSacI-R and the whole length mutant *aroF^fbr ^*gene was obtained. The resulting product was digested with *Sac *I and cloned into pSU2718 cut with the same enzyme, creating pSUaroF^fbr^.

The gene encoding feedback resistant mutant *pheA^fbr ^*G309C [23] was constructed with the primers pheA-M-F/pheA-M-R and pheA-MN-FM/pheA-M-RM using overlap PCR (as described above). The purified PCR fragments of mutant *pheA^fbr ^*were double digested with *Eco*R I and *Hin*d III and then cloned into pKK223-3, creating pKKpheA^fbr^. Then the gene *pheA^fbr ^*with the promoter P*tac *and RBS (ribosome binding site) was amplified from pKKpheA^fbr ^with primers PTacpheA*-F and PTacpheA*-R. The resulting PCR product was ligated into pSUaroF^fbr ^vector, creating pSUFA.

The gene *lacI^q ^*was amplified from pTrc99a with primers lacI^q^-F and lacI^q^-R. Then, it was cut with *Nhe *I and ligated into the same site of pSUFA, creating pSUFAQ.

The gene *hmaS *was amplified from the genomic DNA of *A. orientalis HCCB10007 *(GenBank: HQ679900) using the primers hmaSAo-F and hmaSAo-R. The PCR fragments were digested with *Nde *I and *Hin*d III, and cloned into pET24a vector, generating pEThmaS_Ao_. *hmaS *with the RBS of pET24a was amplified by PCR using primers pETrbs+hmaS_Ao_-F and pETrbs+hmaS_Ao_-R. The corresponding PCR fragments were purified and cut with *Sal *I and *Bam*H I, then cloned into pSUFAQ, generating the recombinant plasmid pSUFAAQ for the production of S-MA (the scheme of plasmid pSUFAAQ can be seen in Figure 2A).

The gene *hmo *was amplified from the genomic DNA of *S. coelicolor *A3(2) M145 (GenBank: AL939115) with the primers hmo-F and hmo-R. The PCR fragments were purified, then digested with *Nco *I and *Kpn *I, and cloned into pTrc99a vector, creating pTrc99ahmo.

The gene *dmd *was amplified from the total cDNA of *R. graminis *ATCC20804 (GenBank: AJ001428) with the primers dmd-F and dmd-R, and cloned into the *Nde *I and *Eco*R I sites of pET24a to create pET24admd. Then, the fragments including *dmd *gene and the RBS of pET24a were amplified with the primers pETrbs+dmd-F and pETrbs+dmd-R. The corresponding PCR fragments were cloned into the *Kpn *I and *Pst *I sites of pTrc99ahmo to generate pTrc99aSD. Then the DNA fragments containing genes *hmo *and *dmd *with P*trc *promoter were obtained by PCR with the primers pTrcSD-F and pTrcSD-R, and inserted into the *Bam*H I site of pSUFAAQ to create pSUFAAQSD for the production of R-MA (the scheme of plasmid pSUFAAQSD can be seen in Figure 2B).

Plasmids were isolated using the AxyPrep Plasmid Miniprep Kit (Axygen, Hangzhou, China). AxyPrep DNA Gel Extraction Kit was used to isolate DNA fragments from agarose gels (Axygen, Hangzhou, China). All PCR fragments were sequencing identified by BioSune (Shanghai, China). DNA polymerase KOD plus for PCR reactions was purchased from ToYoBo (Shanghai, China). All restriction enzymes and Taq DNA polymerase were purchased from Fermentas (Shanghai, China). Rapid DNA ligase and alkaline phosphatase were obtained from TaKaRa (Shanghai, China). Oligonucleotides were ordered from BioSune (Shanghai, China).

The plasmids constructed in this work are listed in Table 1, and the oligonucleotides used are given in Table 2.

**Table 2:** Primers used for gene cloning in this study


**Primer Name**	**Nucleotide Sequence**	**Restriction site**

P148L-F	5'-TTAGATCTGAATAGCCCGCAATACCTGGGC- 3'	
P148L-R	5'-GCTATTCAGATCTAACGCTTCCGTCGCCAGTGG - 3'	
aroFSacI-F	5'-AACGAGCTCACCGGAAAGTCCTCGGGCATAAG - 3'	*Sac *I
aroFSacI-R	5'-AACGAGCTCCGACTTCATCAATTTGATCGCGTAA - 3'	*Sac *I
pheA-M-F	5'-GGGAATTCTATGACATCGGAAAACCCGTTAC - 3'	*Eco*R I
pheA-M-R	5'- ATCCGGAAGCTTTTCATCAGG - 3'	*Hin*d III
pheA-MN-FM	5'- AACAAGCCTGTGCGCTGG - 3'	
pheA-M-RM	5'- TCAACCAGCGCACAGGCTTGTTGC - 3'	
PTacpheA*-F	5'- GATCCGAAGCTTATCGACTGCACG - 3'	*Hin*d III
PTacpheA*-R	5'- AGTCGACGCTTTTCATCAGGTTGG - 3'	*Sal *I
lacI^q^-F	5'- CGCTAGCCCTGACGGGCTTGTCTG - 3'	*Nhe *I
lacI^q^-R	5'- CGCTAGCTTCCGATGGCTGCCTG - 3'	*Nhe *I
hmaSAo-F	5'- CGCAT**ATG**CAGAATTTCGAGATCGACTAC - 3'	*Nde *I
hmaSAo-R	5'- CAAGCTTAACGTACGTCATCGCCG - 3'	*Hin*d III
hmo-F	5'-GATATACC**ATG**GGCAGCAGCCATC-3'	*Nco *I
hmo-R	5'-CGGTACCTGGTCATCCGTGGCTCCTG-3'	*Kpn *I
pETrbs+hmaS_Ao_-F	5'- CCGTCGACAAATAATTTTGTTTAACTTTAAG - 3'	*Sal *I
pETrbs+hmaS_Ao_-R	5'- CGGCCGGATCCTTGAAGATCTC - 3'	*Bam*H I
dmd-F	5'-CCAT**ATG**CCTCGCCCTCGCGTC-3'	*Nde *I
dmd-R	5'-GGAATTCAGTAGGCGCGAAAAGCG-3'	*Eco*R I
pETrbs+dmd-F	5'-CGGTACCTTTGTTTAACTTTAAGAAGG-3'	*Kpn *I
pETrbs+dmd-R	5'-ACTGCAGTGGTGGTGGTGGTGGTGCT-3'	*Pst *I
pTrcSD-F	5'-AGGATCCAAATCACTGCATAATTCG-3'	*Bam*H I
pTrcSD-R	5'-TGGATCCGTTATTGTCTCATGAGCG-3'	*Bam*H I

Underlined text denotes the restriction site sequences, and bold type indicates the start condons.

### Construction of deletion mutants of *E. coli*

The deletion mutants of genes *aspC *and *tyrB *were obtained by a one-step inactivation protocol [32]. The disruption cassettes were amplified from vector pIJ773 or pIJ778 by PCR and then transformed into *E. coli *W3110 that contained the plasmid pIJ790 [33]. The resulting strain with the apramycin resistant cassette inserted into the gene *aspC *site was designated as W3110Δ*aspC*. The *tyrB *deleted mutant W3110Δ*tyrB *was constructed using the spectinomycin resistant cassette by the method described above. Then the multi-deletion mutants were generated by P1 transduction [34] from these single-deletion mutants. The P1 phage lysate prepared from W3110Δ*aspC *was used to infect W3110Δ*tyrB *to produce double-deletion mutant W3110Δ(*tyrB, aspC*). Using the same protocol, we successfully obtained tri-deletion mutants of W3110Δ(*tyrB, aspC, trpE*) and W3110Δ(*tyrB, aspC, tyrA*) by transfer of *trpE *mutation from JW1256-1 or *tyrA *mutation from N3087 using P1 phage to W3110Δ(*tyrB, aspC*), respectively. W3110Δ(*tyrA, tyrB, aspC, trpE*) was generated by transfer of *tyrA *mutation from N3087 to W3110Δ(*tyrB, aspC, trpE*). The disruption cassettes of *aspC::*FRT*-apra-*FRT, *tyrB:: *FRT*-spec-*FRT, *trpE:: *FRT*-kan-*FRT were eliminated by pCP20 [33], except for *tyrA16::Tn10*. Then the resistance markers of W3110Δ*aspC*, W3110Δ*tyrB*, W3110Δ(*tyrB, aspC*), W3110Δ(*tyrB, aspC, trpE*), W3110Δ(*tyrB, aspC, tyrA*), and W3110Δ(*tyrA, tyrB, aspC, trpE*) were removed using FLP-mediated excision of the disruption cassette [33], resulting in strains C, B, BC, BCE, BCA, and BCAE, respectively. All deletion mutants were verified by PCR analysis. All of the *E. coli *mutants constructed in this study are described in Table 1, and the primers used are listed in Table 3.

**Table 3:** Primers used for gene deletion and verification in this study


**Primer Name**	**Nucleotide Sequence**	**Characterization**

tyrB-KO-F	5'-*TTTAACCACCTGCCCGTAAACCTGGAGAACCATCGCGTG*ATTCCGGGGATCCGTCGACC- 3'	Deletion primer
tyrB-KO-R	5'-*ACTGCAGGCTGGGTAGCTCCAGCCTGCTTTCCTGCATTA*TGTAGGCTGGAGCTGCTTC- 3'	Deletion primer
tyrB-V-F	5'- CTGTTGCTAATTGCCGTTCG - 3'	Verification primer
tyrB-V-R	5'- CACGTAGAACGATGGCATCA - 3'	Verification primer
aspC-KO-F	5'-*CGGACTTCCCTTCTGTAACCATAATGGAACCTCGTCATG*ATTCCGGGGATCCGTCGACC- 3'	Deletion primer
aspC-KO-R	5'-*AGCCCGCTTTTCAGCGGGCTTCATTGTTTTTAATGC TTA*TGTAGGCTGGAGCTGCTTC- 3'	Deletion primer
aspC-V-F	5' - CCTGCGTTTTCATCAGTAATAGTTGG - 3'	Verification primer
aspC-V-R	5' - CCTTATCCGGCCTACAAAATCG - 3'	Verification primer
tyrA-V-F	5' - TATCCGTAACCGATGCCTGC - 3'	Verification primer
tyrA-V-R	5' - GGGAAATCACCCGTTCAATG - 3'	Verification primer
trpE-V-F	5' - CGTACTGAAAGGTTGGTGGCG - 3'	Verification primer
trpE-V-R	5' - AGGAGAAAGCATCAGCACCG - 3'	Verification primer

Italics represent the sequences homologous to the gene to be deleted, underlined text denotes the sequence homologous to plasmid pIJ773 or pIJ778 for amplification of the resistance cassette.

### Preparation of cell extract for enzymatic assays

*E. coli *BL21(DE3) strains harboring the plasmids pEThmaS_Ao_, pET24admd, pTrc99ahmo or pTrc99aSD were cultivated in tubes containing 4 mL LB medium with appropriate antibiotics at 37°C at 220 rpm overnight. Then 0.5 mL of culture broth were added to 50 mL of LB medium in 250-mL shake flasks that contained appropriate antibiotics at 30°C at 220 rpm. When OD_600 _reached 0.4~0.6, the cells were induced by addition of 1 mM isopropyl β-D-1-thiogalactopyranoside (IPTG). After 4 h, cells were harvested and washed with 200 mM potassium phosphate buffer (pH 7.5). Then the cell pellet was resuspended in 5 mL 200 mM potassium phosphate buffer (pH 7.5), and the cells were disrupted by French Press (Constant cell disruption systems, United Kingdom) at 25 kpsi. The lysate was centrifuged at 12000 rpm for 20 min (Eppendorf Centrifage 5810R) and the supernatant was used to determine activity. As a control, *E. coli *BL21(DE3) containing plasmid pET24a or pTrc99a was treated in the same manner. The total protein concentration of the supernatant was determined by the Bradford method [35], using bovine serum albumin (BSA) as a standard.

### Enzyme assays

The HmaS assay was performed as described previously [19], with the exception that the 5-mL reaction mixture contained 200 mM potassium phosphate buffer (pH 7.5), 5 mM phenylpyruvate, 44 mM ascorbate, 0.3 mM FeSO_4_, and a final concentration of 1 mg/mL of soluble protein. The reaction was started by the addition of the cell-free extract at 28°C. After addition of 100 μL 1 M HCl to 500-μL samples to stop the reaction, the solution was centrifuged at 12000 rpm for 5 min (Eppendorf Centrifage 5430), and the products were analyzed by HPLC.

The Hmo and DMD assays were performed as described previously [19,20].

### Production of R-MA *in vitro*

In the assay for R-MA production *in vitro*, the 5-mL reaction mixture contained 200 mM potassium phosphate buffer (pH 7.5), 5 mM S-MA, 2 mM NADH, 40 mM NAD^+^, and a final concentration of 1 mg/mL of soluble protein. The reaction was started by the addition of the crude extract at 30°C. Following the addition of 100 μL 1 M HCl to 500-μL samples to stop the reaction, the solution was centrifuged at 12000 rpm for 5 min (Eppendorf Centrifage 5430), and the supernatants were analyzed by HPLC.

### Production of S- or R-MA in *vivo*

The engineered strains BCAE containing the plasmid pSUFAAQ or pSUFAAQSD were inoculated into 4 mL LB medium with chloramphenicol (25 mg/L) and incubated at 37°C at 220 rpm overnight. 4 mL of this culture were subsequently added to 250-mL shake flasks containing 50 mL of the fermentation medium with chloramphenicol (25 mg/L) and incubated at 37°C, 220 rpm. After 4~5 h incubation when the OD_600 _had reached 0.4~0.6, the cells were induced by adding 0.1 mM IPTG, and then the temperature was set to 33°C. Samples were taken at different time points during cultivation and centrifuged at 4°C at 12000 rpm for 5 min (Eppendorf Centrifage 5430). Then the supernatant was frozen at -20°C and prepared for analysis by HPLC, GC, and GC-MS. Cell densities were determined by spectrophotometer OD_600 _(Beckman coulter DU730, USA). Dry cell weight (g/L) was calculated using a conversion coefficient of 0.375 g/L/OD_600_.

### Phenylpyruvate feeding experiments

To test HmaS conversion of phenylpyruvate to S-MA *in vivo*, feeding experiments were performed. The concentration gradient was as follows: 0 mM, 1 mM, 2 mM, 4 mM, and 8 mM. The phenylpyruvate was fed when the OD_600 _reached 0.4~0.6, meanwhile 0.1 mM IPTG was added to the cultures inducing expression of HmaS, and the fermentation temperature was adjusted from 37°C to 33°C for 24 h. Culture samples were analyzed by HPLC.

### HPLC analysis

L-phenylalanine, phenylpyruvate, and S- or R-MA were quantified using a high-performance liquid chromatography system (Agilent Technologies 1,100 series) equipped with a Zorbax Eclipse XDB-C8 column (Agilent, 4.6 mm × 150 mm, 5 μm) at 40°C. The mobile phase consisted of 50 mM phosphate sodium buffer (pH 6.5) and methanol at a volume ratio of 95:5, at a flow rate of 1 mL/min, with UV detection was 215 nm. The pure substances were used as standards.

The S- or R-MA was identified by HPLC with a CHIRALCEL OD-H column (4.6 mm × 250 mm, 5 μm; Daicel Co., Japan) at 30°C. The mobile phase consisted of hexane, isopropanol, and trifluoroacetic acid in a volume ratio of 94:6:0.2, at a flow rate of 1.2 mL/min, and the UV detection wavelength was 228 nm. The pH of samples was adjusted to 1~2 using HCl, then S- or R-MA in the broth was extracted using an equal volume of ethyl acetate.

Glucose concentrations in the broth were determined by HPLC (Model 1200, Agilent) using a Sugar-Pak TM I column (Waters Corp., MA, USA), and the refractive index detector was used [36].

Standard samples were racemic mandelic acid ordered from Fluka, L-phenylalanine from Tokyo Chemical Industry (TCI, Shanghai, China), and phenylpyruvate (sodium), S-MA, and R-MA from Sigma.

### GC analysis

The concentration of acetic acid in the supernatant was determined using gas chromatography (GC) (7890A, Agilent, Wilmington, DE, USA)[36].

### GC-MS analysis

S- or R-MA was further identified by gas chromatography-mass spectrometry (GC-MS). Lyophilized samples were oximated with 20 mg/mL methoxyamine hydrochloride in pyridine at 30°C for 60 min. Then 80 μL pyridine and 20 μL N-methyl-N-[tert-butyldimethylsilyl] trifluoroacetamide were added to the samples for derivatization at 70°C for 30 min. Then 3 μL of derivatized sample were injected for GC-MS analysis after filtration. The GC-MS system (Agilent 6890-5973) was equipped with an HP-5MS column (30 m, 0.25 mm, 0.25 μm), and the electron impact (EI) mode of the mass spectrometer was set to 70 eV. The GC oven temperature was initially held at 60°C and raised with a gradient of 5°C per minute until it reached 180°C. It was then raised with a gradient of 10°C per minute until it reached 260°C. The flow rate of the mobile phase was set to 1 mL/min. All reagents used in this assay were ordered from Sigma.

## Competing interests

The authors declare that they have no competing interests.

## Authors' contributions

SY conceived and supervised the research and revised the manuscript. ZTS designed and performed main experiments, carried out the data analysis and drafted the manuscript. YYN and YML participated in plasmid construction. LXL performed the GC-MS experiments. BBS contributed to enzyme assays. CY contributed to the revision of the manuscript and GC-MS data analysis. WHJ participated in the coordination of this study. All authors read and approved the final manuscript.

## Supplementary Material

Additional file 1 **Chiral chromatography of the MA produced *in vitro***. **(a) **racemic mixture standards; **(b) **BL21(DE3) with pET24a as negative control; **(c) **HmaS stereoconversion of phenylpyruvate to S-MA; **(d) **BL21(DE3) with pTrc99a as negative control; **(e) **Hmo and DMD together transform S-MA to R-MA. Click here for file

Additional file 2 **Chiral chromatography of the MA produced *in vivo***. **(f) **racemic mixture standards; **(g) **the fermentation broth of BCAE with pSUFAQ as negative control; **(h) **S-MA synthesized by strains BCAE harboring pSUFAAQ; **(i) **R-MA synthesized by strains BCAE containing pSUFAAQSD. Click here for file

Additional file 3 **GC-MS analysis of the S-MA produced *in vivo***. S-MA in the broth was further verified by GC-MS: **(a) **the total ion chromatogram of S-MA; **(b) **the m/z profile of S-MA; **(c) **the standard m/z profile of MA from the GC-MS library. Click here for file

Additional file 4 **GC-MS analysis of the R-MA produced *in vivo***. R-MA in the broth was further verified by GC-MS: **(a) **the total ion chromatogram of R-MA; **(b) **the m/z profile of R-MA; **(c) **the standard m/z profile of MA from the GC-MS library. Click here for file

Additional file 5 **Cell densities, acetate and S-MA concentrations in LB medium with glucose**. All single gene mutants and the control strain (BCAE) were cultured in LB medium supplemented with 20 g/L glucose for 48 h. All of the strains harbor the plasmid pSUFAAQ. ND: not detected. Click here for file

Additional file 6 **Cell densities, acetate and S-MA concentrations in fermentation medium**. The triple genes mutant and control strain (BCAE) were grown in fermentation medium for 48 h. Both of the strains harbor the plasmid pSUFAAQ. Data shown are means ± standard deviations, calculated from triplicate individual experiments. Click here for file
