# Supplementary figures and images for: Metabolic engineering of the L-phenylalanine pathway in Escherichia coli for the production of S- or R-mandelic acid
Source: Microb Cell Fact. 2011 Sep 13;10:71. doi: 10.1186/1475-2859-10-71 (PMC3182895; doi:10.1186/1475-2859-10-71)

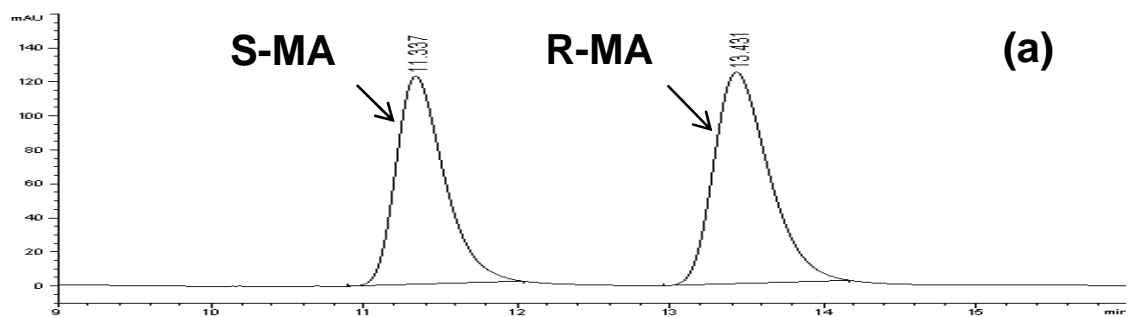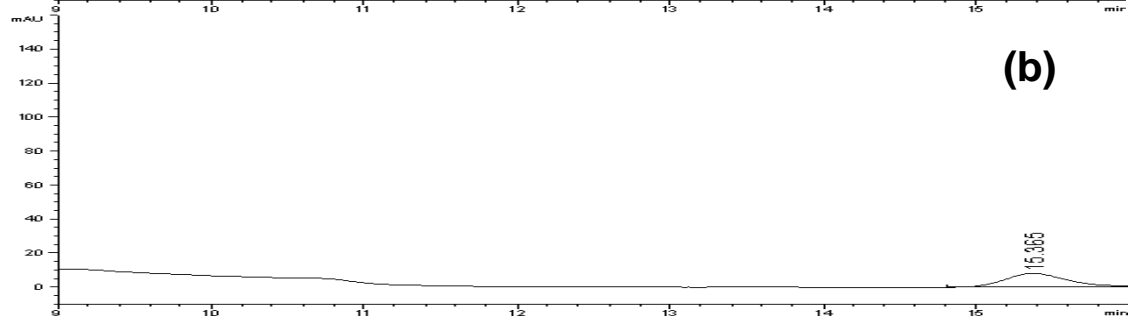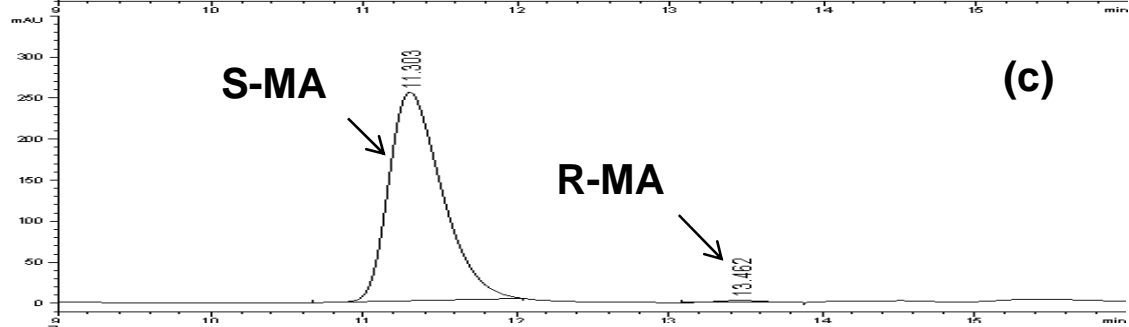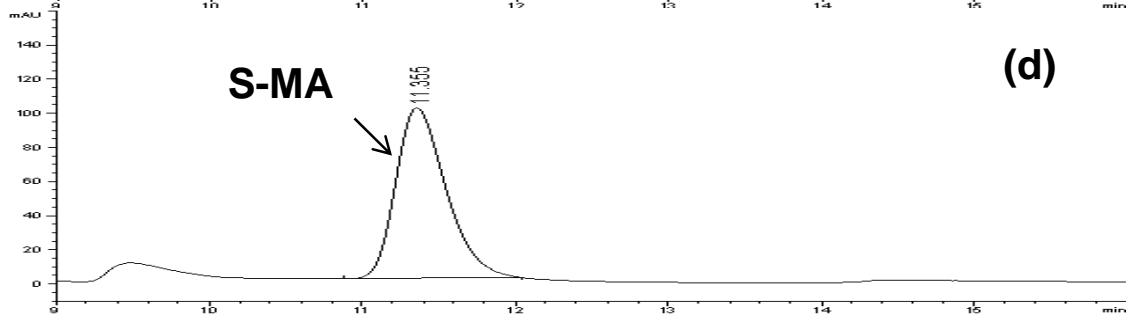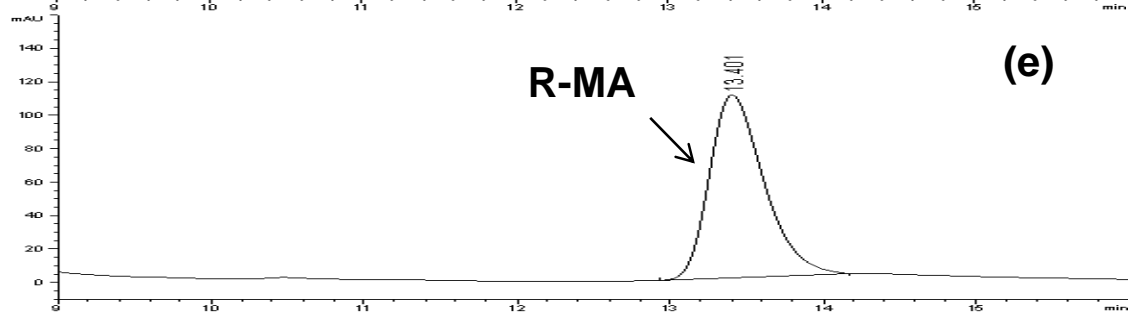

Supplement: Additional file 1 — Chiral chromatography of the MA produced in vitro. (a) racemic mixture standards; (b) BL21(DE3) with pET24a as negative control; (c) HmaS stereoconversion of phenylpyruvate to S-MA; (d) BL21(DE3) with pTrc99a as negative control; (e) Hmo and DMD together transform S-MA to R-MA. [file 1475-2859-10-71-S1.PDF]

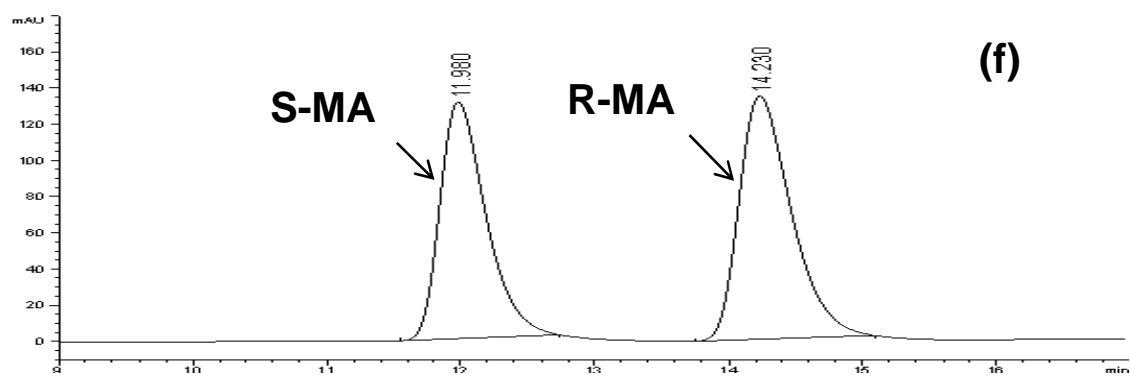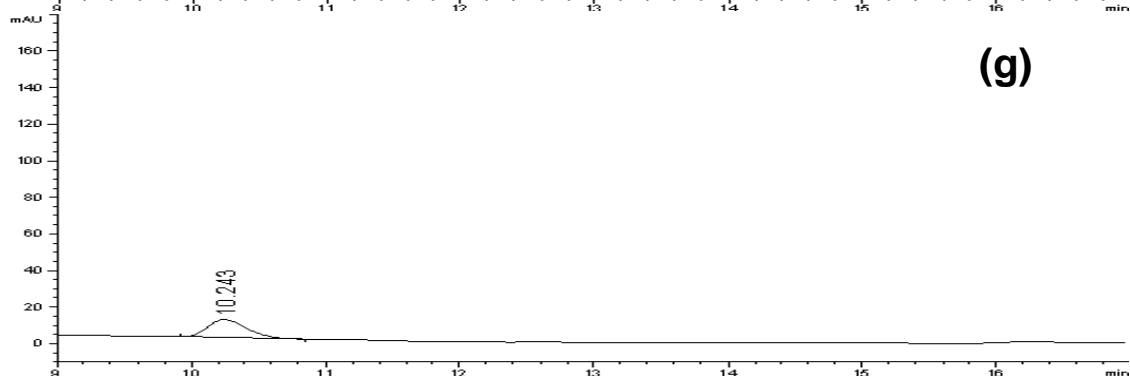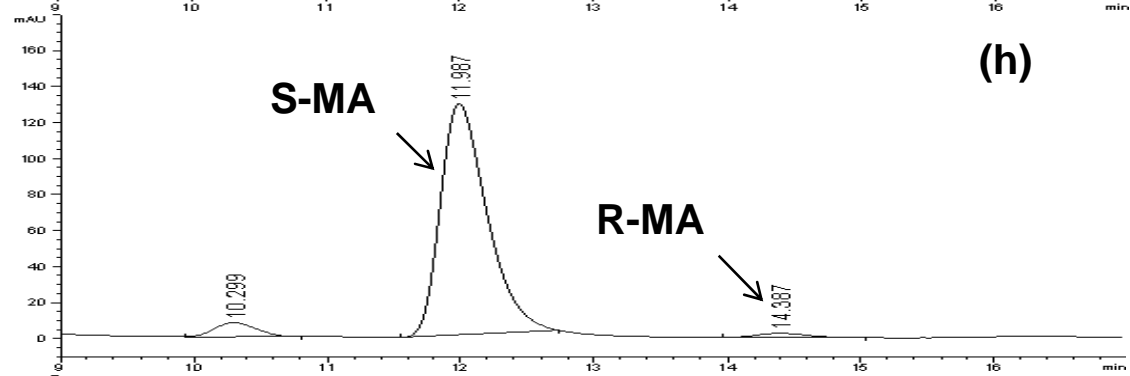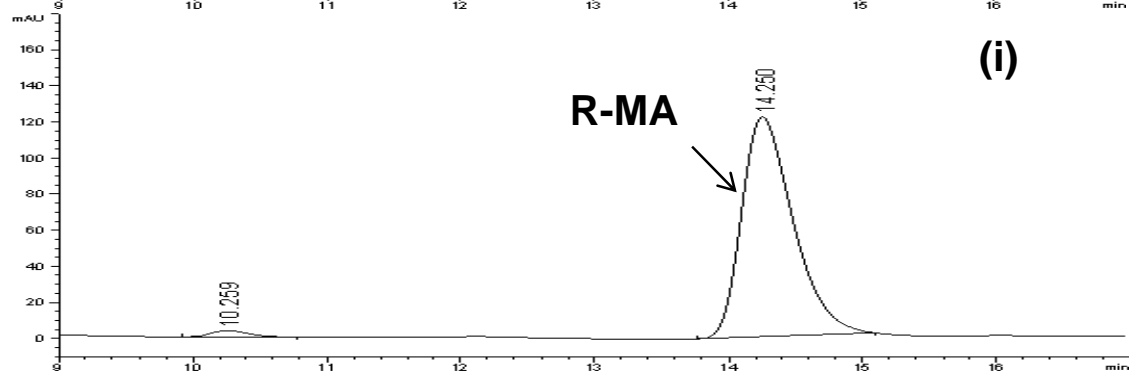

Supplement: Additional file 2 — Chiral chromatography of the MA produced in vivo. (f) racemic mixture standards; (g) the fermentation broth of BCAE with pSUFAQ as negative control; (h) S-MA synthesized by strains BCAE harboring pSUFAAQ; (i) R-MA synthesized by strains BCAE containing pSUFAAQSD. [file 1475-2859-10-71-S2.PDF]

Abundance

TIC: 0201002.D

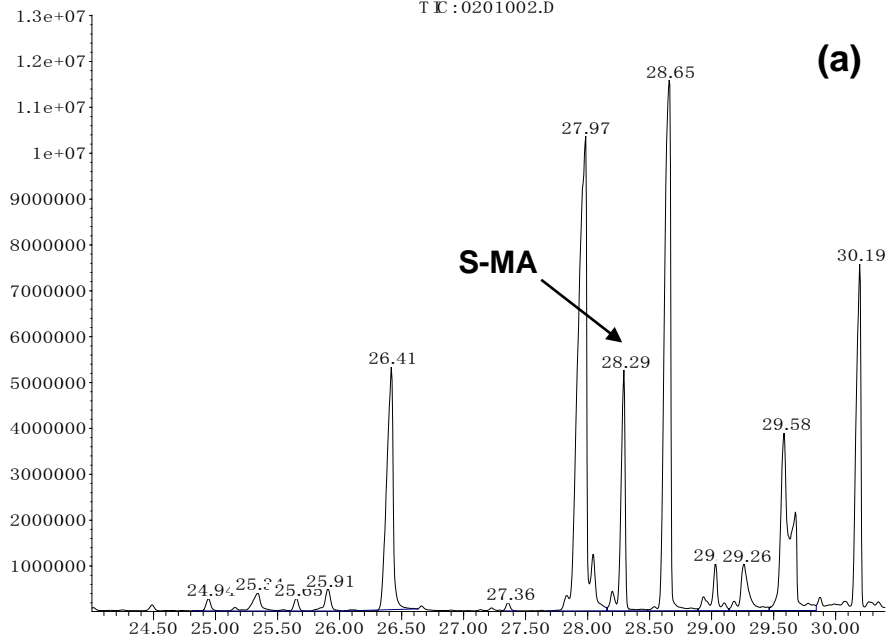

Time→  
Abundance

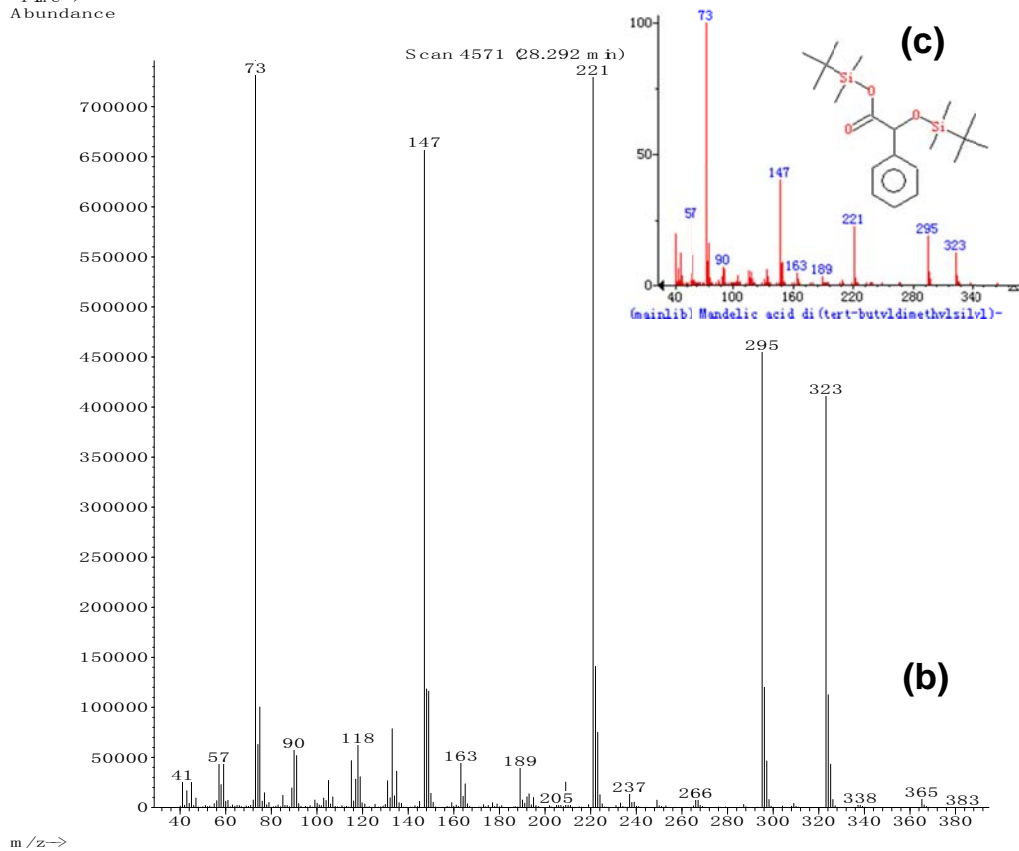

Supplement: Additional file 3 — GC-MS analysis of the S-MA produced in vivo. S-MA in the broth was further verified by GC-MS: (a) the total ion chromatogram of S-MA; (b) the m/z profile of S-MA; (c) the standard m/z profile of MA from the GC-MS library. [file 1475-2859-10-71-S3.PDF]

Abundance

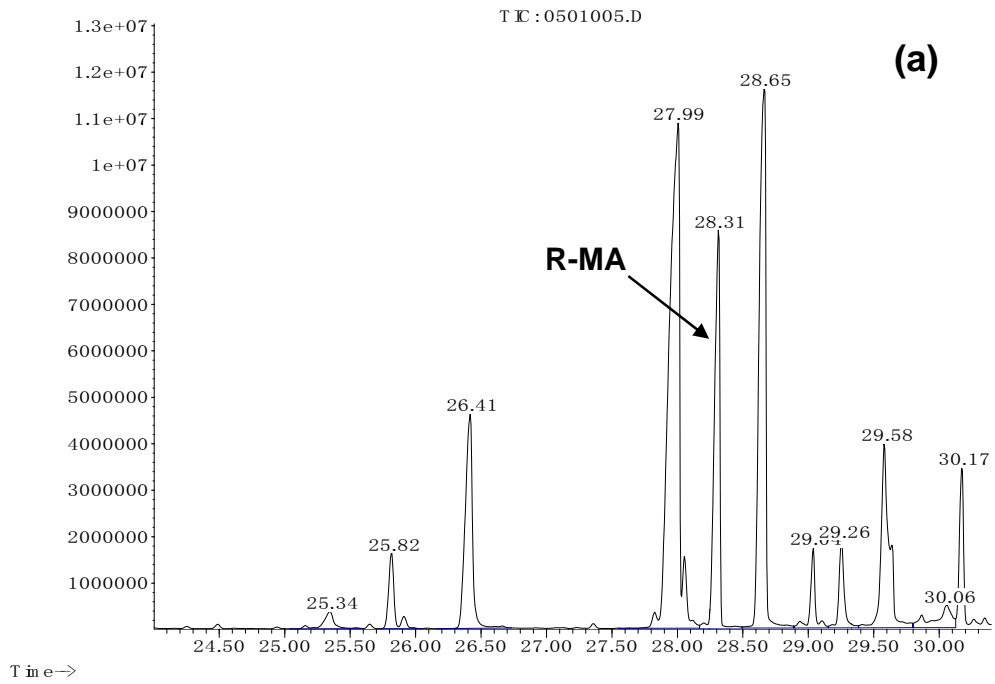

Abundance

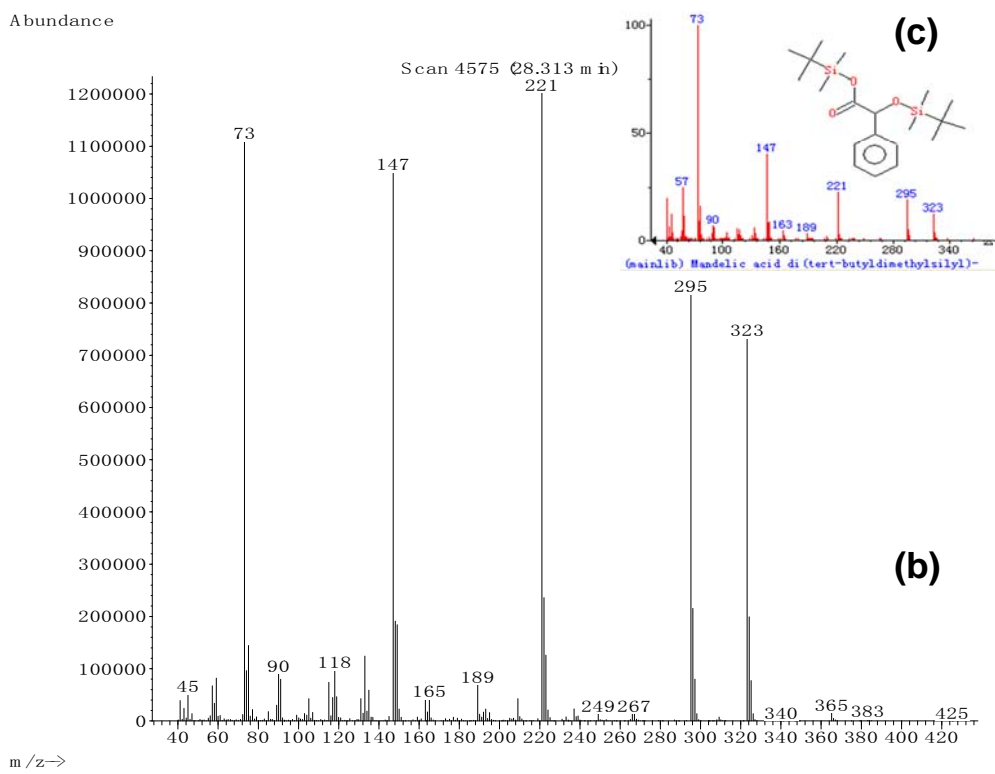

Supplement: Additional file 4 — GC-MS analysis of the R-MA produced in vivo. R-MA in the broth was further verified by GC-MS: (a) the total ion chromatogram of R-MA; (b) the m/z profile of R-MA; (c) the standard m/z profile of MA from the GC-MS library. [file 1475-2859-10-71-S4.PDF]
