# Supplementary material for: Metabolic engineering of the L-phenylalanine pathway in Escherichia coli for the production of S- or R-mandelic acid
Source: Microb Cell Fact. 2011 Sep 13;10:71. doi: 10.1186/1475-2859-10-71 (PMC3182895; doi:10.1186/1475-2859-10-71)
Supplement: Additional file 5 — Cell densities, acetate and S-MA concentrations in LB medium with glucose. All single gene mutants and the control strain (BCAE) were cultured in LB medium supplemented with 20 g/L glucose for 48 h. All of the strains harbor the plasmid pSUFAAQ. ND: not detected. [file 1475-2859-10-71-S5.DOC]

**Additional file 5:** Cell densities, acetate and S-MA concentrations in LB medium with glucose

| Strains | OD_600_ | Acetic acid  (g/L) | S-mandelic acid  (g/L) |
| --- | --- | --- | --- |
| BCAE | 4.99 | 1.66 | 0.535 |
| BCAE ∆poxB | 4.80 | 1.83 | 0.549 |
| BCAE ∆ackA | 4.18 | 1.48 | 0.068 |
| BCAE ∆pta | 3.56 | 1.07 | ND |
| BCAE ∆acs | 5.13 | 1.61 | 0.363 |

All single gene mutants and the control strain (BCAE) were cultured in LB medium supplemented with 20 g/L glucose for 48h. All of the strains harbor the plasmid pSUFAAQ. ND: not detected.
