# Supplementary material for: Metabolic engineering of the L-phenylalanine pathway in Escherichia coli for the production of S- or R-mandelic acid
Source: Microb Cell Fact. 2011 Sep 13;10:71. doi: 10.1186/1475-2859-10-71 (PMC3182895; doi:10.1186/1475-2859-10-71)
Supplement: Additional file 6 — Cell densities, acetate and S-MA concentrations in fermentation medium. The triple genes mutant and control strain (BCAE) were grown in fermentation medium for 48 h. Both of the strains harbor the plasmid pSUFAAQ. Data shown are means ± standard deviations, calculated from triplicate individual experiments. [file 1475-2859-10-71-S6.DOC]

**Additional file 6: Cell densities, acetate and S-MA** concentrations in fermentation medium

| Strains | OD_600_ | Acetic acid  (g/L) | S-mandelic acid  (g/L) |
| --- | --- | --- | --- |
| BCAE | 6.0 ± 0.4 | 4.2 ± 0.3 | 0.92 ± 0.08 |
| BCAE ∆(poxB ackA acs) | 2.7 ± 0.1 | 2.57 ± 0.05 | 0.193 ± 0.007 |

The triple genes mutant and control strain (BCAE) were grown in fermentation medium for 48h. Both of the strains harbor the plasmid pSUFAAQ. Data shown are means ± standard deviations, calculated from triplicate individual experiments.
